# The role of reserve in cognitive and motor function and responsivity to multi‐domain interventions for Mild Cognitive Impairment – Results from the SYNERGIC Study

**DOI:** 10.1002/alz70858_102295

**Published:** 2025-12-25

**Authors:** Eden Mancor, Manuel Montero‐Odasso, Louis Bherer, Quincy J Almeida, Teresa Liu‐Ambrose, Laura E. Middleton, Richard Camicioli, Karen Li

**Affiliations:** ^1^ Concordia University, Montreal, QC, Canada; ^2^ Schulich School of Medicine & Dentistry, Division of Geriatric Medicine, Western University, London, ON, Canada; ^3^ Parkwood Institute, London, ON, Canada; ^4^ Université de Montréal, Montréal, QC, Canada; ^5^ Centre de Recherche de l'Institut Universitaire de Gériatrie de Montréal, Montréal, QC, Canada; ^6^ University of Montreal, Montreal, QC, Canada; ^7^ Care Space Health, Waterloo, ON, Canada; ^8^ University of British Columbia, Vancouver, BC, Canada; ^9^ Schlegel‐UW Research Institute for Aging, Waterloo, ON, Canada; ^10^ University of Waterloo, Waterloo, ON, Canada; ^11^ University of Alberta, Edmonton, AB, Canada

## Abstract

**Background:**

Cognitive and motor deficits have been found to be important markers of Mild Cognitive Impairment (MCI), a pre‐dementia risk state. Recently, aerobic exercise (AE) and cognitive training (CT) interventions significantly improved cognitive and motor function in older adults with MCI. Recently, it has been shown that nearly 50% of dementia cases could be mitigated by the elimination of 12 modifiable risk factors, such as low education and physical inactivity, as early as midlife. Cognitive and Motor Reserve (CR, MR) describe the compensation for cognitive and motor loss through lifelong cognitively and physically enriching experiences, respectively. Our **objectives** are to examine (1) the life historical profiles that contribute to CR and MR, which are ordinarily eliminated in RCTs,(2), if CR and MR predict better cognition and mobility at baseline, and (3) whether they affect responsivity to multi‐domain lifestyle interventions for MCI.

**Method:**

Performing secondary data analysis, participants (*n* = 71) were older adults with MCI randomized to intervention arms: CT+AE, AE only, and control arm. Baseline and post‐intervention assessments of cognition (e.g., executive function, memory) and mobility (simple gait and cognitive‐motor dual tasking) were performed. They also provided historical data on CR and MR factors. We first used principal component analysis (PCA) of the relevant life history variables to calculate weighted CR and MR factors. Second, we ran linear regressions to assess baseline outcomes. Third, we will use linear mixed‐effects (LME) models to examine if CR/MR influence responsivity to the intervention arms.

**Result:**

A larger sample (*n* = 266) revealed two principal components capturing 75.6% of the variance: MR (lifelong physical activity), and CR (education and occupational complexity). Regressions revealed that MR was associated with greater baseline dual‐task walking velocity, and trail‐making task (TMT) performance. CR did not predict baseline scores.

**Conclusion:**

This far, MR predicted baseline cognitive and motor performance. LME will reveal whether MR and CR will impact responsivity to intervention. This far, we conclude that MR may be more sensitive in detecting baseline cognitive and motor benefits. The completed study will illuminate the distinct benefits of CR and MR on intervention efficacy and the relevance of personalized interventions for MCI.

